# Data Harmonization, Standardization, and Collaboration for Diabetic Retinal Disease (DRD) Research: Report From the 2024 Mary Tyler Moore Vision Initiative Workshop on Data

**DOI:** 10.1167/tvst.13.10.4

**Published:** 2024-10-03

**Authors:** Amitha Domalpally, Ward Fickweiler, S. Robert Levine, Kerry E. Goetz, Brian L. VanderBeek, Aaron Lee, Jeffrey M. Sundstrom, Dorene Markel, Jennifer K. Sun

**Affiliations:** 1 Wisconsin Reading Center, University of Wisconsin, Madison, WI, USA; 2 Department of Ophthalmology, Harvard Medical School, Boston, MA, USA; 3 Mary Tyler Moore Vision Initiative, Greenwich, CT, USA; 4 Office of Data Science and Health Informatics, National Eye Institute, National Institutes of Health, Bethesda, MD, USA; 5 Scheie Eye Institute, Department of Ophthalmology, University of Pennsylvania Perelman School of Medicine, Philadelphia, PA, USA; 6 Department of Ophthalmology, University of Washington, Seattle, WA, USA; 7 Department of Ophthalmology, Penn State College of Medicine, Hershey, PA, USA; 8 Department of Ophthalmology and Visual Sciences, Kellogg Eye Center, University of Michigan Medical School, Ann Arbor, MI, USA; 9 University of Michigan Department of Learning Health Sciences, Ann Arbor, MI, USA

**Keywords:** data standardization, diabetic retinopathy, data harmonization

## Abstract

The 2024 Mary Tyler Moore Vision Initiative (MTM Vision) Workshop on Data convened to discuss best practices and specific considerations for building a comprehensive, shareable MTM Vision data lake. The workshop aimed to accelerate the development of new indications, therapies, and regulatory pathways for diabetic retinal disease (DRD) by standardizing and harmonizing clinical data and ocular ’omics analyses. Standardization of data collection, the use of common data elements, and data interoperability were emphasized, alongside federated learning approaches to promote data sharing and collaboration while maintaining data privacy and security. The integration of molecular data with other multimodal data types was recognized as a promising strategy for leveraging machine learning and AI approaches to advancing therapeutics development and improving treatment outcomes for DRD patients. Partnerships with entities such as the National Eye Institute, part of the National Institutes of Health, foundations, and industry were deemed vital for the successful implementation of these initiatives.

## Introduction

Mary Tyler Moore (1936–2017) was a renowned actress who used her iconic status to help raise awareness of the devastating consequences of diabetes and its complications and the promise of research. Diagnosed with type 1 diabetes at age 33, she became International Chairman of Juvenile Diabetes Research Foundation (now Breakthrough T1D) in 1984 and, in partnership with her husband, Dr. S. Robert Levine, tirelessly advocated for funding of diabetes research, helping to secure billions of dollars directed to the cause of finding better treatments and cures. The Mary Tyler Moore Vision initiative (MTM Vision) was launched to honor her and focus attention on the global health challenge of vision loss and blindness caused by diabetic retinal disease and, ultimately, to help realize her dream of a world without vision loss from diabetes.

The mission of MTM Vision is to accelerate the development of new methods to preserve and restore vision in people with diabetes. To foster collaboration and gather insights from key leaders in this multidisciplinary field, MTM Vision has organized semi-annual workshops. These workshops are designed to shape and guide the trajectory of the MTM Vision, ensuring a cohesive and forward-thinking approach to catalyze breakthroughs by eliminating barriers to progress and providing tools and globally accessible resources linked to collaborative networks sharing their data. MTM Vision's Fall 2022 Workshop on clinical endpoints focused on identification of the most promising biomarkers that need further development and validation for diabetic retinal disease (DRD).^1^ This report will provide a synopsis of the Spring 2024 Mary Tyler Moore Vision Initiative Workshop on Data that focused on best practices and specific considerations for building a large, shareable MTM Vision data lake, including methods for clinical data standardization and harmonization of ’omics analyses to accelerate the development of new indications, therapies, and regulatory pathways for DRD.

## MTM Vision Roadmap

S. Robert Levine, MD, the founder and CEO of the Mary Tyler Moore Vision Initiative (MTM Vision), set the stage by refreshing the audience with MTM Vision's Roadmap, which was published earlier.^1^ MTM Vision aims to accelerate the development of new methods to preserve and restore vision for people with diabetes. Phase 1 of the MTM Vision Roadmap emphasizes overcoming barriers to progress in this field via three principal projects: (1) updating the staging system and severity scale for DRD; (2) establishing a biorepository of ocular tissues and fluids to study human diseases; and (3) assessing promising endpoints in DRD to improve diagnosis and care and facilitate new drug development via informing of new regulatory pathways. The DRD staging system update project will add patient perspectives, measures of visual function, and retinal physiology, serologic, and other clinical data variables including prospective surveillance to advanced imaging data and to deploy AI tools to create a multidimensional and highly personalized assessment of patient status, disease severity, and risk of progression to enhance clinical decision-making, and patient selection for treatment and research. Workgroups were established to focus on key components of a DRD staging update, including diabetic retinal vascular disease,^2^ retinal neuronal disease,^3^ basic and cellular mechanisms^4^ systemic factors,^5^ visual function,^6^ and quality of life.^7^

To facilitate the study of DRD in humans, MTM Vision has established a biorepository at the University of Michigan that is collecting, characterizing, and storing ocular tissues and fluids that are shared with other investigators. To better understand the disease at cellular and molecular levels, identify therapeutic targets, and validate biomarkers for DRD, multi-omics analysis is being performed, and the data from these analyses will be shared with qualified researchers on a dedicated platform.

 To achieve the goals of the MTM Vision s DRD Endpoints project, two clinical studies have been proposed. The first is an observational natural history study of retinal function in 400 participants with diabetes and varying stages of DRD over four years. The second proposed study is an observational longitudinal study of retinal function in about 100 patients treated for diabetic macular edema with anti-vascular endothelial growth factor (anti-VEGF) agents over one year. The primary goal of these studies is to develop primary endpoints of visual function that are acceptable for use in clinical trials for regulatory approval. A secondary goal is to validate biomarkers as surrogate clinical endpoints that can be used to enhance research and improve clinical care. A major component of these studies will be development of patient-reported outcomes, aligning with the mission of MTM Vision to seek the insights of individuals personally affected by diabetes and DRD. For example, a gait assessment pilot is planned because changes in gait parameters appear to be associated with visual field loss.^8^

The second phase of the MTM Vision Roadmap focuses on advancing diagnostics and therapeutic target identification through the use of large integrated datasets from diverse populations. This will facilitate the development of AI-based systems for improved diagnosis, risk prediction, and clinical outcomes for patients with DRD. A therapeutic target identification core will be established, modeled after successful initiatives such as the University of Michigan's Kidney Translation Core and Renal Pre-Competitive Consortium, to support global precision-medicine research and industry collaborations.^9^ The phased MTM Vision approach aims to establish a diagnostic basis for new indications for treatment of DRD, to identify new therapeutic targets at the molecular and cellular level, and to inform new regulatory pathways directed to new indications, ultimately improving clinical assessment and care for patients with DRD. The creation of a cross-sector consortium will facilitate access to biospecimen and data assets, accelerating progress in this field. Data harmonization and standardization are critical components to promote this collaboration.

## Data Standardization and Interoperability in Ophthalmology Research

Michael Chiang, MD, Director of the National Eye Institute (NEI), part of the National Institutes of Health (NIH), discussed collaborative efforts in standardization of data to facilitate the establishment of datasets for research. Multiple organizations such as NEI, the Food and Drug Administration (FDA), the Office of the National Coordinator of Health Information Technology, the American Academy of Ophthalmology, and the Association of Research and Vision in Ophthalmology have endorsed this data standardization paradigm.^10^^–^^13^ Modern research in ophthalmology increasingly relies on big data, necessitating data sharing to overcome the limitations of smaller studies. Emphasizing the importance of clinical data interoperability, Dr. Chiang advocated for the adoption of common data models such as Observational Medical Outcomes Partnership (OMOP) to create an integrated data ecosystem with standardized cohorts and phenotypes (Fig.).^14^ Incentives for data sharing and data standardization are being promoted by journals like such as *Translational Vision Science and Technology*, which has established a Data Science section and publishes Data Science descriptor articles featuring scientifically valuable data sets and software libraries relevant to all aspects of vision science.^14^ In addition, NEI and NIH are exploring novel approaches to complement the NIH data management and sharing policy and promote a culture of team science.^15^ The future research paradigm should move toward large datasets and collaborative efforts, valuing “knowledge” over individual journal articles and encouraging the vision research community to lead this transformation.

**Figure.**
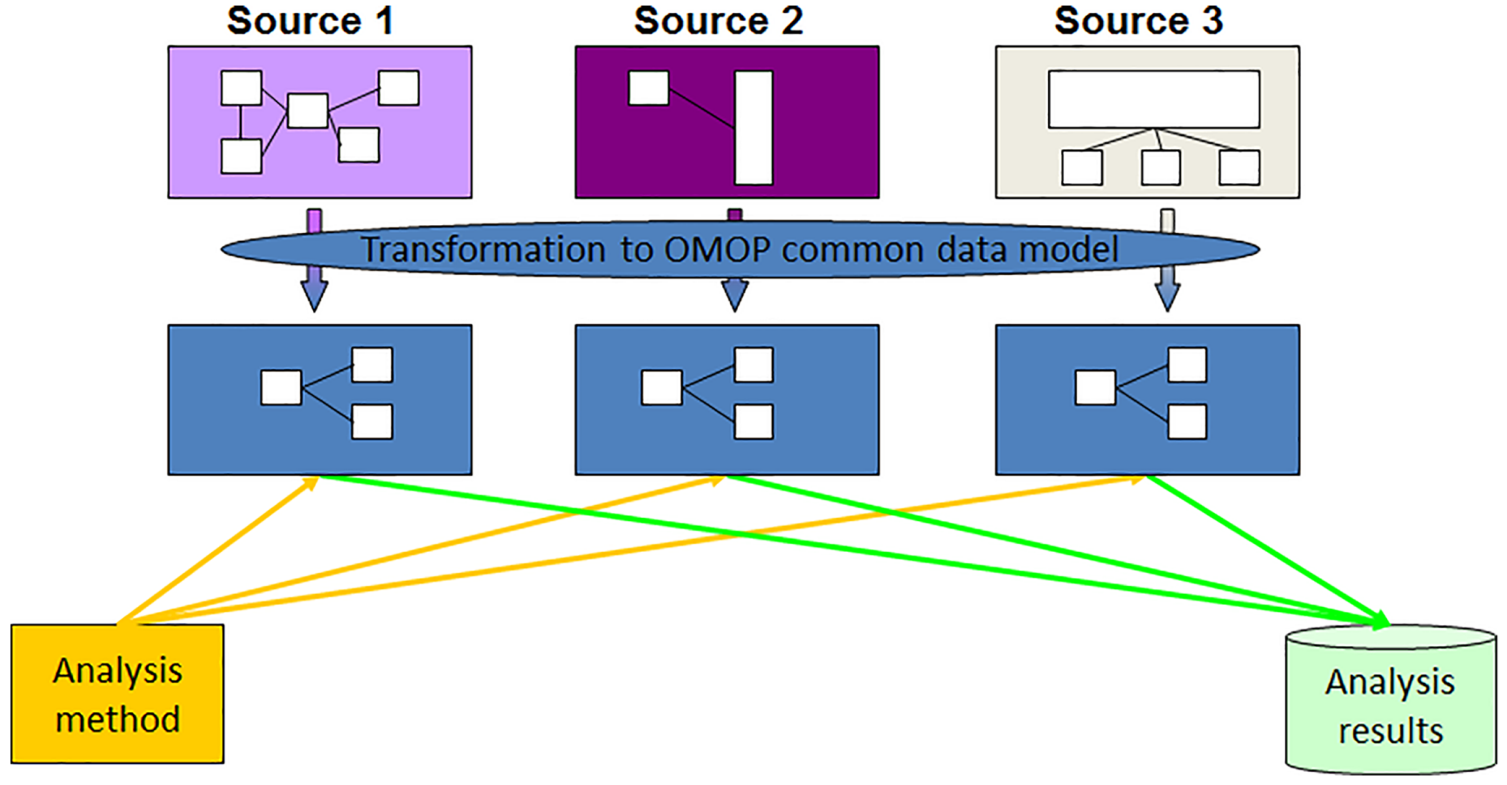
The diagram illustrates the transformation process from multiple data sources into the OMOP Common Data Model. Source 1, Source 2, and Source 3 represent different datasets, each with unique structures. These datasets are standardized and mapped into the OMOP Common Data Model, ensuring consistent data representation. Once transformed, the harmonized data is subjected to analysis methods, resulting in unified analysis results. This standardization facilitates the integration and comparison of data from diverse sources for robust clinical and epidemiological research.

Kerry Goetz, MS, NEI Office of Data Science and Health Informatics, expanded on approaches for data standardization methods including data collection and organization. Centralized data collection, as exemplified by the Intelligent Research In Sight (IRIS) Registry,^16^ the Sight Outcomes Research Collaborative (SOURCE) Consortium (https://ihpi.umich.edu/our-experts-partners/collaborating-centers-programs/CEPI), All of Us^17^ and National COVID Cohort Collaborative^18^ involves aggregating data from multiple sites into a single repository. Although this approach facilitates easy access and analysis, it requires extensive legal, privacy, and security agreements, as well as significant resources to harmonize and maintain the data.

Another approach to data sharing, which reduces the burden of data sharing agreements and privacy concerns is via federated learning. Federated data networks, such as the Observational Health Data Sciences and Informatics,^19^ National Pediatric Learning Health System,^20^ and European Health Data Evidence Network, map local data from institutions within the network to a common data model, which facilities local query and analysis without the need for the data to leave the sources. Alignment to an agreed upon data model allows for modern research approaches (including machine learning) while preserving data-privacy through distributing model training and aggregating model weights without sharing individual patient/participant data.^21^ This method supports scalability, large imaging, and the inclusion of both ocular and systemic data but necessitates substantial effort and resources at each site for data standardization and analysis.

Common data elements are critical to enabling interoperability and analytics on disparate data. Common data elements provide a combination of precisely defined questions paired with a specified set of responses common to multiple datasets or studies.^22^ Standardizing data elements into case report forms is crucial for comparing research studies from many sources. This standardization is essential for making datasets findable, accessible, interoperable, and reusable^23^ and can facilitate making large data fully AI-ready while also enhancing the quality and efficiency of clinical research.

Table 1 outlines some organizations involved in harmonization of local elements to data models, stewardship of data standards, and system interoperability standards and explains the datasets on which these standards can be applied. This list is not exhaustive.

**Table 1.** Healthcare Data Standards and Models for Clinical Research and Regulatory Frameworks

Organization/Collaborative Groups	Standards/Models	Description	Example
Observational Health Data Sciences and Informatics	OMOP	A common data model that standardizes **diverse health data** (e.g., claims, health records, etc.) for large-scale **research** analytics	Using OMOP to integrate data from multiple hospitals to analyze risk of kidney failure with anti-VEGF exposure^24^
CDISC (Clinical Data Interchange Standards Consortium)	CDISC Standards	Develops global data standards to streamline **clinical trial data** and ensure data interoperability and FDA preferred submission format	Conducting a multi-center clinical trial for a new diabetic retinopathy drug, ensuring data consistency across sites using CDISC standards
HL7 (Health Level Seven International)	FHIR (Fast Healthcare Interoperability Resources)	A standard for **electronic health information exchange** and analytics using EHR data	Seamlessly transferring a patient's detailed DRD treatment history from a primary care physician to a specialist using FHIR
DICOM (Digital Imaging and Communications in Medicine)	DICOM (Digital Imaging and Communications in Medicine)	A standard for handling, storing, transmitting, and viewing **medical imaging** information	Using DICOM standards to store, retrieve, and share OCT scans from multiple machines in a vendor neutral imaging platform
LOINC (Logical Observation Identifiers Names and Codes) Regenstrief Institute	LOINC (Logical Observation Identifiers Names and Codes)	A clinical terminology and international standard for coding and describing clinical and laboratory observations	Recording and sharing standardized lab results, such as HbA1c levels, across different healthcare systems using LOINC codes
SNOMED International (Systematized Nomenclature of Medicine)	SNOMED CT (Systematized Nomenclature of Medicine Clinical Terms)	A comprehensive **clinical healthcare terminology** including diseases, symptoms, procedures, and other medical concepts	Documenting patient records with standardized terms for various stages of DRD and macular edema, using SNOMED CT
WHO (World Health Organization)	ICD (International Classification of Diseases)	A **diagnostic tool** for classification of diseases. Primarily used for insurance reimbursement and for public health tracking	Healthcare providers use ICD codes to classify and report cases of diabetic retinopathy; e.g. ICD-10-CM Diagnosis Code E11. 329: Type 2 diabetes mellitus with mild non proliferative diabetic retinopathy without macular edema
AMA (American Medical Association)	CPT (Current Procedural Terminology) Codes	Provides codes for **medical procedures** and services. Primarily used for insurance reimbursement	Coding procedures like retinal laser therapy or intravitreal injections for diabetic retinopathy treatment using CPT codes

## Addressing Data Gaps and Enhancing Standardization in Diabetic Retinopathy Research

The presentation by Brian VanderBeek, MD, MPH, MSCE, University of Pennsylvania Scheie Eye Institute, emphasized the critical need for data standardization in DRD research. One of the key concerns highlighted was the inconsistency and incompleteness of crucial clinical data for DRD research across large databases. For instance, hemoglobin A1c (HbA1c) data is often lacking or incomplete. For example, the IRIS Registry and Medicare have no HbA1c data, whereas insurance databases like Optum and Truven have partial data for about 25% of diabetic patients. Health system registries like SOURCE and TriNetX have higher rates of HbA1c data but still suffer from significant missingness because of the open nature of these systems. In addition, the other primary predictor of DRD progression, duration of diabetic disease, is not contained within any of the above databases.

To address these gaps, VanderBeek advocates for the use of the Diabetes Complications Severity Index,^25^ a validated metric using ICD codes to categorize diabetes complications, including retinopathy, nephropathy, neuropathy, cerebrovascular, cardiovascular, peripheral vascular disease, and metabolic conditions like ketoacidosis. The Diabetes Complications Severity Index has been shown to better predict clinical outcomes, such as hospitalization and death, than either disease duration or HbA1c alone.^26^ The comprehensive inclusion of such data will enhance the usability of real-world data for DRD research, providing a more holistic view of patient health and improving the accuracy of predictive models for disease progression and outcomes.

## Bridge2AI Data Standardization Project

The Bridge2AI initiative, funded by the NIH, aims to accelerate the widespread adoption of AI in biomedical and behavioral research by creating high-quality, ethically sourced datasets.^27^ Aaron Lee, MD, MSCI, University of Washington, overall principal investigator of one of the six Bridge2AI initiatives, presented the Salutogenesis Data Generation, also known as the Artificial Intelligence Ready and Equitable Atlas for Diabetes Insights (AI-READI) project (https://aireadi.org/). Salutogenesis is a social science theory that studies the origins of health and well-being, rather than the causes of disease.^28^ The goal of the AI-READY project is to create a multidimensional, ethically sourced dataset in diverse people for studying salutogenesis in Type 2 Diabetes. This triple balanced dataset for age, gender, and race includes a wide range of data types, such as survey responses, clinical measurements, retinal images, laboratory results, ECG data, physical activity levels, genetic sequences, and environmental factors. By integrating diverse data sources, the project seeks to provide a robust foundation for AI-driven insights and equitable healthcare solutions.

The initial installment of data has been released and involves 204 participants, generating substantial data, including 6,841 2D macular and optic disc images, 774,047 optical coherence tomography (OCT) B-scan images, 505,264 OCT angiography flow B-scan images, and extensive systemic measures and physical activity data amounting to 311 GB of data.

The AI-READI project is focused on preparing an AI-ready dataset through the integration of various data types using standardized formats as shown in Table 2.

**Table 2.** Standardization of Datasets From Various Sources in the BRIDE2AI AI-READY Project

Data Class	Data Type	Standardization Format
Structured data	Demographics, Survey data, Physical assessment data, Medications, Blood and urine lab values, MOCA, Vision testing	OMOP
Imaging	Fundus photography, OCT, OCTA, FLIO	DICOM
Cardiology	ECG	Waveform Database
Monitoring data	Physical activity monitor, Continuous glucose monitor	mHealth
Environmental data	Environmental sensor	Earth Science Data Specification

This extensive collection will support the development of AI tools and models to improve diabetes care and outcomes. By promoting data sharing and standardization, the AI-READI project aims to foster a collaborative research environment, driving innovation in the understanding and treatment of Type 2 diabetes.

### Panel Discussion

The panel discussion included Aaron Lee, MD, MSCI, Sally Baxter, MD, MSc, University of California San Diego; Drew Lewis, Co-Founder/President Estenda Solutions; and Paolo Silva, MD, Joslin Diabetes Center; and was moderated by Jennifer Sun, MD, MPH, Science Co-Director of MTM Vision. It focused on key considerations for building a data lake covered several key themes. First, the importance of data quality and regulatory compliance was highlighted. Standardized annotation of raw data and labeling by reading centers are essential for usability. Although clinical trial data is crucial to include in this data lake, real-world data, despite its imperfections, is indispensable for real-world implementation. The importance of large datasets, the impact of data source and firmware variability, and the need for comprehensive longitudinal data were underscored as essential considerations for effective data use. Last, the discussion on data infrastructure focused on the advantages and challenges of centralized versus federated data models. Federated learning was recognized for its potential in enhancing privacy and facilitating data sharing across institutions, although it requires significant local computational resources and expertise. The complexities of setting up federated networks, the need for standardization, and the extended time required to establish agreements and infrastructure were highlighted. Central governance and structured protocols were recommended to ensure consistency and efficacy in data management across federated networks.

## Standardization of Ocular Tissues and Fluid

Jeffrey M. Sundstrom, MD, PhD, Pennsylvania State University College of Medicine, presented the process of standardization of sample collection, characterization, storage, and analyses for ocular tissues and fluids. The vitreous biorepository project of MTM Vision aims to collect and annotate vitreous samples for molecular analysis to identify pathways involved in ocular diseases and to use data to validate pre-clinical models for drug development. Since its inception in 2012, the biorepository at the University of Michigan has collected approximately 3500 samples, with 90% of annotations completed. This resource provides investigators with access to well-annotated samples to study various ocular diseases, enhancing the development of targeted therapies. The collection process follows a standard operating procedure (SOP) that involves gathering 400 µL of undiluted vitreous fluid from patients, splitting this into two tubes, and storing them carefully in −80°C freezers. Biorepository curation SOPs are also being used for annotation of each sample by ocular disease and other relevant clinical information to ensure that future studies and investigators can select relevant samples.

The second aim of this project is to develop methods for optimal proteomic analysis of vitreous fluid from surgical samples and apply vitreous proteomics to provide insights into retinal diseases. Specific goals include to validate vitreous as proximal biofluid for retinal disease and to conduct power analysis to verify validity of pathway analysis. A small-scale proteomic study at Michigan^29^ identified upregulation in angiogenesis and significant neurodegenerative components in the vitreous of PDR patients. Other studies have also suggested that the vitreous proteome can reflect retinal disease processes including the detection of retinol binding protein 3 in ocular fluids and VEGF in ischemic ocular diseases in ocular fluids.^30^ Initially, the cost of mass spectrometry for proteomic analysis posed a significant challenge, but advancements in multiplex analysis have since reduced costs and increased the depth of protein coverage that can be achieved. Recent studies identified mor than 3500 different proteins in vitreous samples, and ongoing analysis aims to refine these findings to identify the most promising biomarker or biomarker panels for DRD. Multiplex analyses are planned to include 50 to 200 candidate biomarkers that will then be narrowed down to three to 10 clinical biomarkers that are related to DRD. The development of a cost effective and safe method to acquire vitreous in clinical settings would enable efficient collection of thousands of vitreous samples, which are essential for validating biomarkers in large-scale studies. By integrating discovery proteomics methods with clinical data, the project also seeks to create companion diagnostics for ocular diseases, ultimately improving patient outcomes. Advanced techniques which include Olink proteomics are being employed to increase the depth of proteome coverage, furthering the potential for new discoveries in the molecular mechanisms of retinal diseases and DRD.^31^

### Panel Discussion

The panel discussion on “Data Standardization” with Kerry Goetz, MS; Brian VanderBeek, MS, MPH, MSCE, Michelle Hribar, PhD, Oregon Health & Science University; and Emily Chew, MD, NEI; and moderated by Thomas Gardner, MD, MS, Science Co-Director MTM Vision; and the panel discussion on “Standardization of Sample Collection, Characterization and Storage and ’Omic Analysis of Ocular Tissues and Fluids” with Jeffrey M. Sundstrom, MD, PhD; Vinit Mahajan, MD, PhD, Stanford Medicine; Stephen Kim, MD, Vanderbilt University Medical Center; and Remko Bakker, PhD, Boehringer Ingelheim, underscored the need for enhanced accessibility to ocular tissues, integration of multi-omics data with clinical and imaging data, and implementation of robust data-sharing practices for achieving breakthroughs in the understanding and treatment of DRD. Establishing centralized biobanks that process and store ocular fluids and tissues from various donors and institutions can enhance the quality and reproducibility of omics analyses by using standardized and consistent protocols. Additionally, fostering collaborations between academic institutions, hospitals, and pharmaceutical companies can facilitate the sharing of these valuable resources. Ethical frameworks and regulatory policies need to be standardized globally to streamline the process of tissue collection and distribution. Another significant opportunity lies in the integration of multi-omics data with clinical and imaging data and advanced machine learning techniques. Combining molecular data sets with clinical and imaging data can greatly enhance patient phenotyping, possibly leading to more personalized and effective treatments in DRD. The inclusion of collecting human fluids and tissues in randomized controlled trials can lead to a more precise categorization of patients based on their responsiveness to treatments by characterizing molecular biomarkers, advancing our understanding of the underlying molecular pathways in the development and progression of DRD.

## Opportunities for Collaboration

Collaboration between MTM Vision with NIH, FDA, foundations, international organizations, and industry is critical to successfully build a large, shareable MTM Vision data lake. Examples of successful data sharing models were discussed, including the Diabetic Retinopathy Clinical Research Retina Network for clinical trial data, the Oracle Learning Health Network, IRIS registry, UK Biobank, and the Helmsley charitable trust funded T1D Exchange data^32^ for real-world data. These data consortia illustrate the potential benefits of standardized data collection and sharing, which can lead to more efficient and impactful research in ophthalmology.

### Panel Discussion

The panel including Thomas Brunner, President and CEO, Glaucoma Research Foundation; Tunde Peto, MD, PhD, Queen's University of Belfast and Ulrich Luhmann, PhD, Roche Innovation Center; and moderated by S. Robert Levine, MD, emphasized that collaboration and effective data sharing requires investments in a reliable IT infrastructure and clear frameworks to manage competitive interests and ensure that data sharing initiatives are aligned with common goals between different entities. In addition, it is critical for the MTM Vision data lake to collaborate with patients early in the process and communicate with patients about how their data will be used. This transparency is also crucial in building trust and ensuring patient willingness to contribute their data. Although data sharing is becoming more accepted among newer researchers, significant barriers remain, particularly within academic institutions that prioritize individual achievements such as first authorship over collaborative success. There is a need for cultural and structural changes to facilitate better data sharing practices and a shift toward a more collaborative culture in science supported by policies and incentives from funding agencies, which include the NIH/NEI. Practical steps such as providing basic analysis tools and creating common data elements can make data more accessible and usable, thus fostering a more collaborative research environment. It is important to start with practical, manageable projects to build momentum and demonstrate success, with the recognition that initial efforts might not be perfect but are essential for progress for creating a shareable MTM Vision data lake.

## Summation

MTM Vision is dedicated to accelerating research in DRD to preserve and restore vision in patients with diabetes with the ultimate goal of a world without vision loss in patients with diabetes. Key to this is the creation of a comprehensive MTM Vision data lake, supported by data sharing and clinical interoperability. The data lake will integrate diverse data sources while maintaining privacy and security, promoting a collaborative research environment. The concept of building a data lake was highlighted through the AI-READI project, which is a remarkable example of a large, shareable data repository. The shareable MTM Vision data lake should prioritize data cleanliness, data standardization, and identifying and prioritizing key variables that should be consistently collected from both EHR and clinical trial data. This requires a well-trained informatics IT workforce and infrastructure capable of managing and analyzing large datasets across different institutions while at the same time ensuring patient privacy and controlled access. The power and importance of utilizing human vitreous for translational DRD studies highlighted the need for human ocular samples in advancing these studies. The combination of molecular data with other multimodal data types for machine learning and AI approaches holds promise for augmenting the precision and efficacy of treatments in patients with DRD.

Incentivizing data sharing by institutions like the NIH and academic journals, alongside public and private sector collaboration, is important. Several barriers to collaboration include the necessity for a sufficiently long timeframe to enable meaningful collaborations to develop, the importance of obtaining appropriate informed consent from patients, and the establishment of a precompetitive space to facilitate collaborative efforts. Despite these challenges, there is an ongoing cultural shift toward a greater willingness to collaborate, reflecting a growing recognition of the benefits of shared efforts in advancing scientific knowledge to preserve or restore vision in patients with diabetes.

Several key next steps include identifying data sources, establishing SOPs for data acquisition, standardization, and sharing, and building support through the MTM Vision Consortium. Partnerships with entities like NEI, foundations, and industry are vital. By leveraging diverse stakeholders and fostering collaboration, MTM Vision aims to significantly advance DRD understanding and treatment, ultimately achieving a world without vision loss for people with diabetes.
